# *Porphyromonas gingivalis*-Induced MIF Regulates Intercellular Adhesion Molecule-1 Expression in EA.hy926 Cells and Monocyte-Endothelial Cell Adhesion Through the Receptors CD74 and CXCR4

**DOI:** 10.1007/s10753-018-0942-0

**Published:** 2018-12-03

**Authors:** Yun Wu, Wanyue Xu, Jingya Hou, Yanqing Liu, Rong Li, Jingbo Liu, Chen Li, Xiaolin Tang, Li Lin, Yaping Pan, Dongmei Zhang

**Affiliations:** 1 Department of Periodontics, School of Stomatology, China Medical University, Heping District, Nanjing North Street No.117, Shenyang, 110002 China; 2 Department of Periodontics and Oral Biology, School of Stomatology, China Medical University, Heping District, Nanjing North Street No.117, Shenyang, 110002 China

**Keywords:** *Porphyromonas gingivalis*, atherosclerosis, macrophage migration inhibitory factor, intercellular cell adhesion molecule-1, CD74, CXCR4

## Abstract

*Porphyromonas gingivalis* (*P. gingivalis*) is an important pathogen that contributes to periodontal disease and causes infections that promote the progression of atherosclerosis. Our previous studies showed that macrophage migration inhibitory factor (MIF) facilitates monocyte adhesion to endothelial cells by regulating the expression of intercellular adhesion molecule-1 (ICAM-1) in*P. gingivalis-*infected endothelial cells. However, the detailed pathological role of MIF has yet to be elucidated in this context. To explore the functional receptor(s) of MIF that underlie its participation in the pathogenesis of atherosclerosis, we investigated the expression of the chemokine receptors CD74 and CXCR4 in endothelial cells, both of which were shown to be involved in the adhesion of monocytes to endothelial cells pretreated with *P. gingivalis*. Furthermore, the formation of a MIF, CD74, and CXCR4 ligand-receptor complex was revealed by our immunofluorescence staining and coimmunoprecipitation results. By interacting with the CD74/CXCR4 receptor complex, MIF may act as a crucial regulator of monocyte-endothelial cell adhesion and promote the atherosclerotic plaque formation induced by *P. gingivalis*.

## INTRODUCTION

Periodontitis is one of the most prevalent infectious diseases in the human oral cavity. As a major periodontal pathogen, the presence of *Porphyromonas gingivalis* (*P. gingivalis*) in atherosclerotic lesions suggests an association between periodontitis and atherosclerosis [1, 2], a relationship that has been confirmed by epidemiological data, clinical studies, and animal experiments [3–7]. In addition,*in vitro* research has shown that *P. gingivalis* can increase the expression of cell adhesion molecules, proinflammatory cytokines, and chemokines in endothelial cells, which have crucial roles in the recruitment of monocytes to the vascular endothelium and the subsequent formation of atherosclerotic plaques [8–12].

The recruitment and adhesion of monocytes *via* the synergistic responses of multiple chemokines and their receptors has been shown to be crucial events underlying atherosclerotic lesion formation and disease progression [13, 14]. As a highly conserved and atypical proinflammatory cytokine with chemokine-like functions, macrophage migration inhibitory factor (MIF) exerts multipotent immune functions in chronic inflammatory diseases, such as rheumatoid arthritis, atherogenesis, and cancer [15–17]. MIF has been demonstrated to primarily promote atherosclerosis through the enhancement of macrophage and T cell recruitment by directly affecting endothelial-monocyte interactions [18, 19].

We previously reported that *P. gingivalis* infections enhance endothelial MIF and intercellular adhesion molecule-1 (ICAM-1) expression, in addition to promoting the adhesion of monocytes to endothelial cells [20]. Furthermore, we demonstrated that the increased adhesive properties induced by*P. gingivalis* were dependent on MIF expression [21]. Our findings suggested that *P. gingivalis* infections lead to endothelial activation and pro-atherosclerotic lesion formation. During this inflammatory process, MIF may undertake a regulator role in monocyte recruitment and atherogenesis.

MIF mediates cellular responses and triggers several signaling pathways by binding to its receptors [22, 23]. Although advances have recently been made in understanding how *P. gingivalis* promotes atherosclerosis [2, 24], a detailed understanding of how the activities of MIF and its functional receptors participate in atherosclerotic diseases remains unclear. In this study, we investigated potential MIF receptors that facilitate ICAM-1 expression and monocyte adhesion to endothelial cells to provide new insights into the pathogenesis of *P. gingivalis*-promoted atherosclerosis. The results of our study revealed the molecular mechanism of MIF regulation of monocyte-endothelial cell adhesion. We demonstrated that MIF is a functional ligand of chemokine receptors CD74 and CXCR4 and that it participates in the regulation of monocyte recruitment in atherosclerosis promoted by *P. gingivalis* infection.

## MATERIALS AND METHODS

### Cells

EA.hy926 cells (a human umbilical vein endothelial cell line) and THP-1 cells (a monocyte cell line) were used in our study, both of which were acquired from Keygen Biotech Company (Nanjing, China). EA.hy926 cells were maintained in Dulbecco’s modified Eagle medium (DMEM; Gibco BRL, Carlsbad, CA, USA) supplemented with 15% fetal bovine serum (FBS; GeneTimes, Shanghai, China), and THP-1 cells were grown in DMEM containing 10% FBS. Both cell lines were cultured at 37 °C with 5% CO_2_. A trypan blue exclusion test was used to assess cell viability. The EA.hy926 cells were used in the following assays when the observed cell viability was > 90%. Before the two cell lines were co-cultured, the fluorescent dye calcein-AM (0.1 mg/mL; BioVision, Bay Area, CA, USA) was used to label the THP-1 cells in the dark for 30 min.

### Bacterial Strain

*P. gingivalis* ATCC 33277 was routinely maintained in brain heart infusion broth supplemented with 5% defibrinated sheep’s blood, 0.5% yeast, 0.1% menadione, and 1% hemin and was cultured under anaerobic conditions (80% N_2_, 10% O_2_, and 10% H_2_) at 37 °C. The bacterial cells were collected, and the optical density of the bacterial suspension was adjusted to 1.0 at 600 nm before infecting EA.hy926 cells.

### Analysis of CD74 and CXCR4 Expression by Western Blot

EA.hy926 cells were infected with *P. gingivalis* at a multiplicity of infection (MOI) of 100 for 24 h, after which the expression of CD74 and CXCR4 was assessed by Western blot. Cells cultured without *P. gingivalis* were used as a negative control.

After the cells were lysed, the protein concentration in cell lysates was determined by a BCA assay. The samples were separated by 10% SDS-PAGE and transferred to a nitrocellulose membrane, with GAPDH used as a loading control. After blocking, proteins of interest were detected with specific primary antibodies, including a mouse anti-CD74 mAb (1:500; Santa Cruz Biotechnology, Santa Cruz, CA, USA), a mouse anti-CXCR4 mAb (1:500; Proteintech, Rosemont, IL, USA), and a mouse anti-GAPDH antibody (1:1000; Wanlei, Shenyang, China). After an overnight incubation, the blots were washed and then incubated with Dylight 800 conjugated rabbit anti-mouse IgG (1:1000; Abbkine, Inc., Redlands, CA, USA) for 1 h. Odyssey CLX (LI-COR, Lincoln, NE, USA) was exploited for Western blot analyses. The relative protein expression levels were presented.

### Analysis of ICAM-1 Protein and Gene Transcription by Western Blot and qRT-PCR

Endothelial cells were pretreated with a neutralizing antibody of CD74 (C-16, 5 μg/mL; Santa Cruz Biotechnology) [22, 25], an inhibitor of CXCR4 (AMD3100, 20 nM; Abcam, Cambridge, MA, UK) [22, 25] or DMEM medium for 1 h. Next, the cells were infected by*P. gingivalis* for 24 h (MOI = 100). The cells treated with culture medium only were used as a control. Then, the whole cell protein was extracted and samples were analyzed for ICAM-1 expression by Western blot as described above using rabbit anti-ICAM-1 mAb (1:500; Wanlei, Shenyang, China) and Dylight 800 conjugated goat anti-rabbit IgG (1:1000; Abbkine, Inc.) antibodies.

Using cells that were treated as described above, a quantitative real-time polymerase chain reaction (qRT-PCR) assay was performed as described in our previous study [21]. Briefly, TRIzol reagent (Invitrogen, Carlsbad, CA, USA) was utilized to extract total cellular RNA, the purity of which was evaluated by determining the 260/280 nm absorbance ratio. Biosystems 7500 Fast Real-Time PCR System (RR047, RR420, Takara, Tokyo, Japan) was used to analyze the ICAM-1 mRNA expression, together with the SYBR® Premix Ex Taq™ II (RR047, RR420, Takara, Tokyo, Japan), which was used according to the manufacturer’s protocol. The following primers were used for qRT-PCR: ICAM-1 forward: 5′-TGATGGGCAGTCAACAGCTA-3′, ICAM-1 reverse: 5′-GCGTAGGGTAAGGTTCTTGC-3′, GAPDH forward: 5′-GAAGGTCGGAGTCAACGGAT-3′, GAPDH reverse: 5′-CCTGGAAGATGGTGATGGGAT-3′. The primers of ICAM-1 and GAPDH were designed by Primer 3, and the specificity was verified by blasting primer sequences against the NCBI database. The mRNA level of the internal reference GAPDH was designed as 100%, and *ICAM-1* mRNA is presented compared to the GAPDH reference.

### Adhesion Assays of THP-1 to EA.hy926 Cells

Endothelial cells were grown on six-well plates until a confluent monolayer was formed, after which the cells were treated as described above. Later, THP-1 cells (1 × 10^6^, labeled with 5 μM calcein-AM) were co-cultured with endothelial cells for an additional 1 h at 37 °C with 5% CO_2_ keeping in the dark_._ Next, the non-adherent THP-1 cells were rinsed with PBS. After being fixed with a 4% formaldehyde solution, the labeled monocytes adhered to the surface of EA.hy926 cells were examined with a fluorescence microscope (Nikon 80i, Tokyo, Japan). Three fields were chosen randomly and the adherent monocytes were identified through visual cell counting.

### Immunofluorescence Staining and Colocalization Analysis

Endothelial cells were cultured on cover slips in 24-well plates at a concentration 4 × 10^4^ cells per well for 24 h at 37 °C. Next, the cells were infected with *P. gingivalis* at an MOI of 100 for 24 h. The cells were subsequently washed and fixed, after which they were incubated in 1% BSA in 0.1% PBS-Tween for 1 h. Afterwards, the samples were incubated with mouse anti-CD74 (1:100; Santa Cruz) and rabbit anti-CXCR4 (1:200; Abcam) overnight at 4 °C. Donkey anti-mouse IgG labeled with PE (1:20; Proteintech) and goat anti-rabbit IgG labeled with FITC (1:20; Proteintech) were used as the secondary antibodies and were incubated with the cells for 1 h at room temperature. DAPI (Boster, Wuhan, China) was used to stain the cell nucleus (pseudo-colored blue), and the cells were observed using a fluorescence microscope (Nikon 80i, Japan) to evaluate the colocalization between CXCR4 and CD74.

### Transfection Assays of EA.hy926 Cells

The plasmid pGCsi-H1 Neo GFP (Genechem, Shanghai, China) was used to construct a stably transfected EA.hy926 cell line in which *CXCR4* was silenced*.* shRNA oligonucleotide fragment interference vectors with interference segment information 5′-GGGUGUGAGUUUGAGAACA-3′ were established, and cells transfected with empty vectors were used as a negative control. EA.hy926 cells in logarithmic growth phase were transfected using lipofectamine 2000 (Invitrogen) as a transfection reagent. Pure cell lines were obtained by selection with G418 (500 μg/ml; Invitrogen). The cells were incubated for 48 h, and the total cellular CXCR4 content was determined by anti-CXCR4 Western blot and qRT-PCR to assess the knock down efficiency. The positive monoclonal cell line was cultured in DMEM containing 15% FBS at 37 °C with 5% CO_2_ and was used in subsequent assays.

### Coimmunoprecipitations and Pull-Down Experiments

EA.hy926 cells stably transfected with the *CXCR4*-silencing plasmid, normal EA.hy926 cells, and the negative control transfected cells were infected with *P. gingivalis* at an MOI of 100 for 24 h. Next, whole cell protein was extracted from the samples. After diluting the cell proteins with PBS to 1 μg/μl, samples were incubated with a rabbit anti-MIF mAb (1 μl/500 μl; Abcam) or an IgG control overnight at 4 °C for immunoprecipitation. Protein A agarose beads were washed twice with PBS and then were diluted to a concentration of 50% by PBS. Next, the prepared protein-A agarose beads and the lysates were incubated together for 2 h at 4 °C, after which the beads bound with the immune complexes were washed three times with pre-chilled PBS. The beads were subsequently resuspended in sample buffer and boiled, and then the boiled samples subsequently used for Western blot to assess the presence of MIF, CXCR4, and CD74. Blots were obtained by using rabbit anti-MIF mAb (1:1000; Abcam), mouse anti-CD74 mAb (1:500; Santa Cruz), and mouse anti-CXCR4 mAb (1:500; Proteintech). To ascertain uniformity of protein loading, the membrane was immunoblotted with anti-GAPDH (1:1000; Wanlei). Goat anti-rabbit or rabbit anti-mouse Dylight 800 conjugated IgG (1:1000; Abbkine, Inc.) were used as secondary antibodies and were incubated with the blots for 1 h at room temperature. Lysates without immunoprecipitation were tested by Western blot as controls.

### Statistical Analysis

Data are presented as the means ± SD of three repeated experiments. Student’s *t* test in SPSS 17.0 was used to evaluate the significant differences between the groups. Differences with a*P* < 0.05 were considered significant.

## RESULTS

### *P. gingivalis* Infection Induces the Expression of CXCR4 but Not CD74 in Endothelial Cells

We previously reported that *P. gingivalis* infection facilitates the expression of ICAM-1 and the adhesion of THP-1 and EA.hy926 cells by upregulating the secretion of MIF [21]. These findings prompted us to investigate the mechanisms by which MIF promotes the adhesion of the endothelial cells, which are closely associated with the inflammatory process of atherosclerosis.

**Fig. 1:**
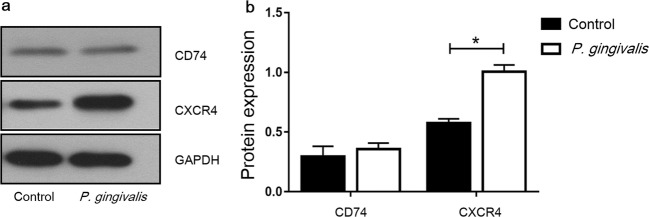
*P. gingivalis* infection induced the protein level of CXCR4 but not CD74 in EA.hy926 cells. *P. gingivalis* infected EA.hy926 cells for 24 h at MOI = 100, then the expression of CD74 and CXCR4 was detected by Western blot. Cells cultured without *P. gingivalis* were used as a control. **a** Western blot analysis of CD74 and CXCR4 in endothelial cells. **b** Quantitative analysis of the Western blot. Data were presented compared to the GAPDH reference. *P < 0.01.

To evaluate the involvement of possible MIF receptors in this process, we examined the expression of CD74 and CXCR4 in EA.hy926 cells. The Western blot results showed that the expression of CXCR4 but not CD74 in EA.hy926 cells was induced by *P. gingivalis*. Compared to the un-infected control cells, CXCR4 expression was increased 1.75-fold by *P. gingivalis* infection (24 h, MOI = 100; *P* < 0.01). In contrast, CD74 expression remained unchanged after *P. gingivalis* infection (Fig. 1).

### *P. gingivalis* Induction of ICAM-1 and*ICAM-1* mRNA Expression Is Partially Dependent on CD74 and CXCR4

**Fig. 2:**
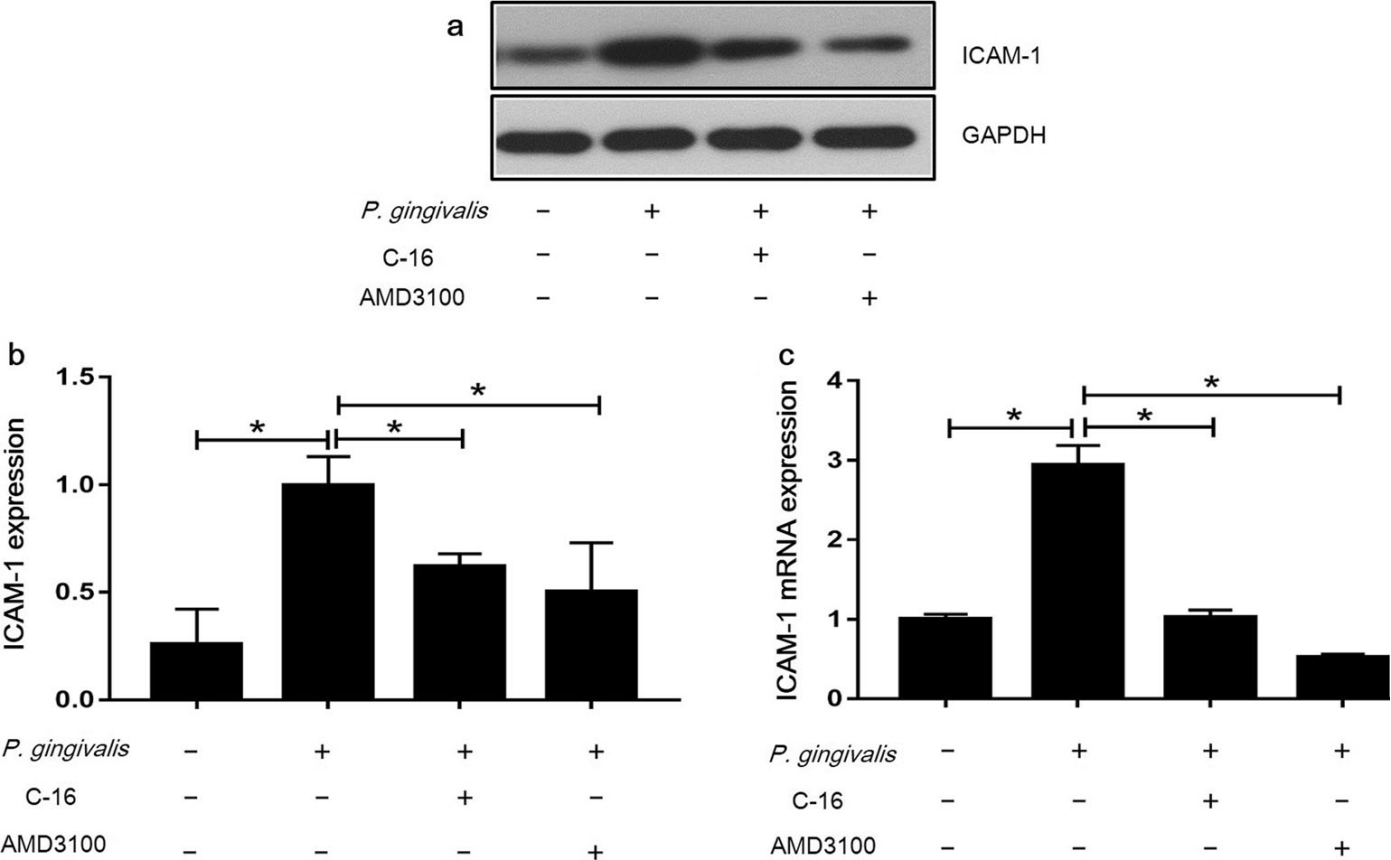
*P. gingivalis* induction of ICAM-1 and *ICAM-1* mRNA expression is partially dependent on CD74 and CXCR4. EA.hy926 cells were infected with *P. gingivalis* at MOI = 100 for 24 h after the addition of C-16 or AMD3100, then the level of ICAM-1 and *ICAM-1* mRNA was determined by Western blot and qRT-PCR. EA.hy926 cells cultured in medium only were used as a negative control. **a** Western blot analysis of ICAM-1.**b** Quantitative analysis of Western blot. **c** Quantitative real-time PCR analysis of I*CAM-1* mRNA. *P < 0.01.

*P. gingivalis* infection was previously shown to enhance *ICAM-1* mRNA and protein expression in endothelial cells. In this study, we explored the roles of the receptors CD74 and CXCR4 on changes in ICAM-1 levels. We analyzed the expression of ICAM-1 after specifically blocking CD74 or CXCR4. A neutralizing CD74 antibody (C-16) and a CXCR4 inhibitor (AMD3100) were individually added to EA.hy926 cells for 1 h before being treated with *P. gingivalis*. Our results demonstrated that the expression of ICAM-1 was significantly upregulated by *P. gingivalis* (Fig. 2a–c), and this induction by *P. gingivalis* was counteracted by the neutralizing antibody of CD74 or the CXCR4 inhibitor treatments. Compared with the cells infected by *P. gingivalis* alone, C-16 or AMD3100 reduced the ICAM-1 protein level by 38% (*P* < 0.01) and 50% (*P <* 0.01), respectively (Fig. 2a, b). Further confirmation of these results was obtained through our qRT-PCR findings. The expression profile of *ICAM-1* mRNA was consistent with that of ICAM-1 protein levels, as *ICAM-1* mRNA levels were reduced by 66% (*P <* 0.01) and 82% (*P <* 0.01) by the addition of C-16 or AMD3100, respectively (Fig. 2c). It was notified that the *ICAM-1* mRNA level in the AMD3100 treatment group was lower than the control. But the statistical analysis showed there was no significant difference between the two groups. Our results indicated that the induction of ICAM-1 and *ICAM-1* mRNA in endothelial cells by *P. gingivalis* was partially dependent on CD74 and CXCR4.

### Enhanced Adhesion of Monocytes to *P. gingivalis*-Infected Endothelial Cells Is Partially Dependent on CD74 and CXCR4

**Fig. 3:**
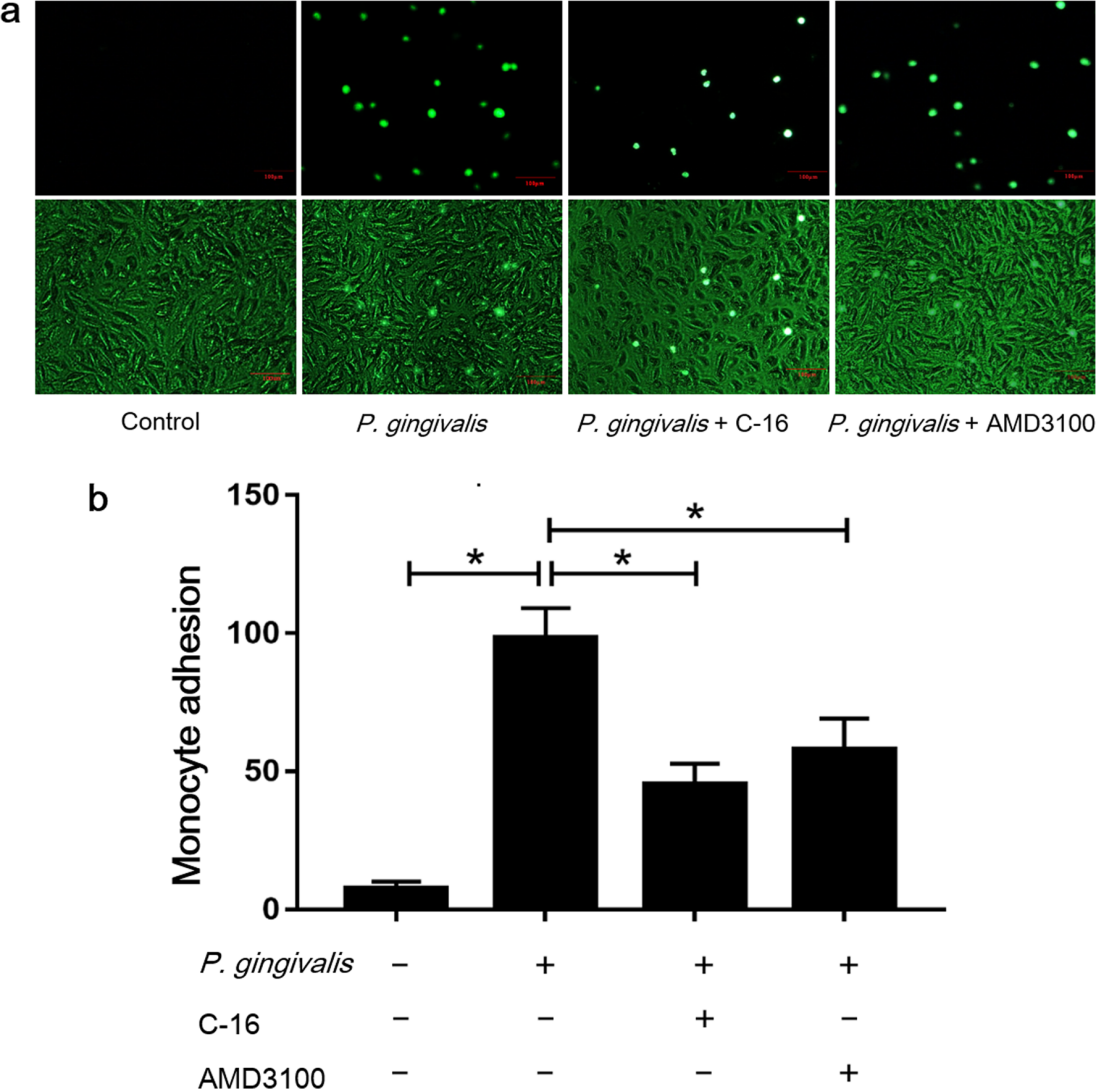
Enhanced THP-1 cells adhesion to *P. gingivalis*-infected EA.hy926 cells is partially dependent on CD 74 and CXCR4. The EA.hy926 cells were pre-incubated with C-16 (5 μg/mL) or AMD3100 (20 nM) for 1 h then infected with *P. gingivalis* for 24 h (MOI = 100). THP-1 cells labeled with Calcein-AM (5 μM) were co-cultured with EA.hy926 cells for additional 1 h before the adhesion assay. The control group was EA.hy926 cells pre-treated with culture medium only.**a** Calcein-AM labeled THP-1 cells adhered to EA.hy926 cells under fluorescence microscope (upper) or microscope (lower) (magnification × 100). Representative pictures were captured in three independent experiments. **b** Cell count assay to evaluate the adherent THP-1 cells. *P < 0.01. Scale bar = 100 μm.

Our early assays indicated that the enhanced adhesion of monocytes to endothelial cells induced by *P. gingivalis* was regulated by MIF. In this assay, we evaluated the possible roles of the receptors CD74 and CXCR4 in the observed monocyte-endothelial cell adhesion. Bacterial infection has been previously observed to increase cell adhesion by 8.17-fold [21]. Compared to the endothelial cells infected with *P. gingivalis*, the C-16 and AMD3100 pretreatments reduced monocyte adhesion by 54% (*P <* 0.01) and 41%, respectively (*P <* 0.01; Fig. 3). Thus, the results of this assay showed that the *P. gingivalis*-induced promotion of monocyte-endothelial cell adhesion is also dependent on CD74 and CXCR4.

### CD74 and CXCR4 Colocalize in EA.hy926 Cells Infected with *P. gingivalis*

**Fig. 4:**
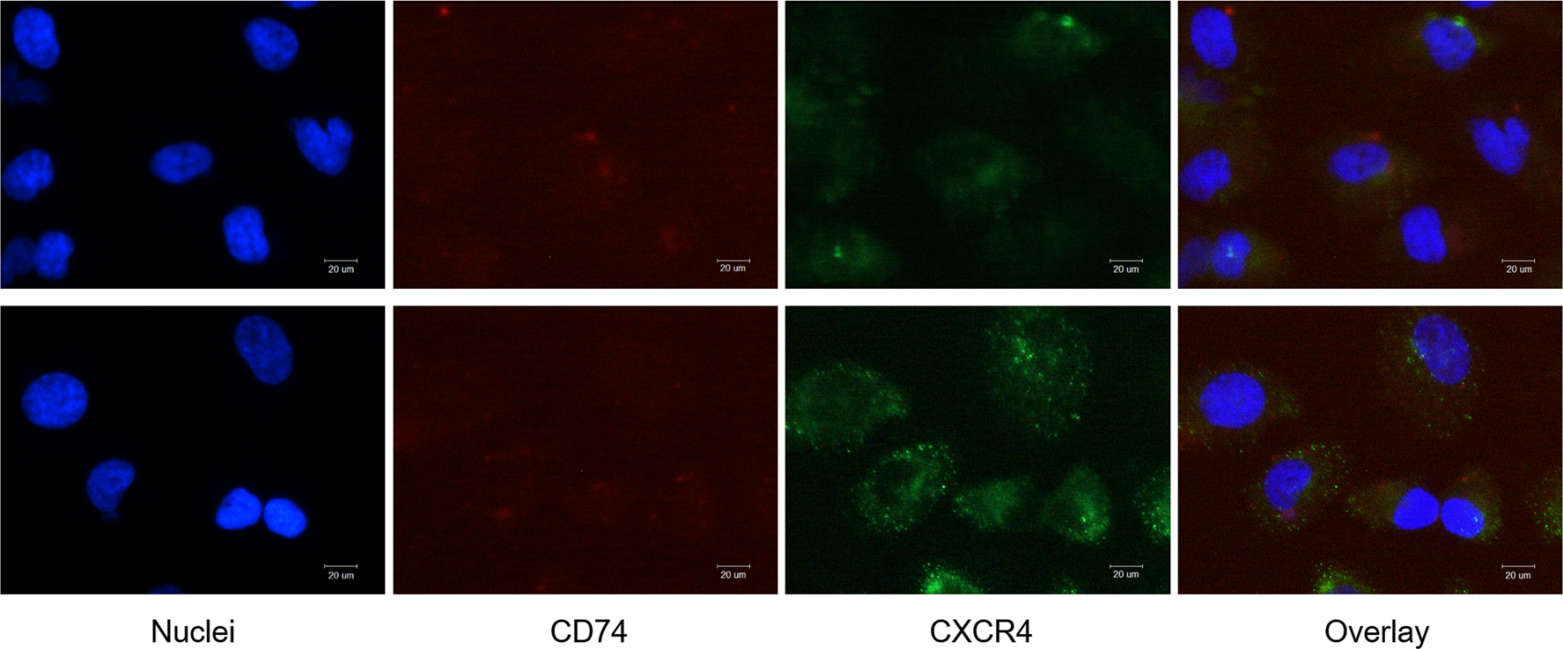
Colocalization of CD74 and CXCR4 in EA.hy926 cells infected with *P. gingivalis*. EA.hy926 cells infected with *P. gingivalis* (24 h, MOI = 100) were observed by fluorescence microscopy (bottom). The cells cultured with medium alone were used as a control (top). Colocalization of CD74 and CXCR4 in the plasma membrane (orange-yellow overlay) was shown. After three independent experiments, representative pictures were captured by fluorescence microscope (magnification × 400). Scale bar = 20 μm.

CD74 and CXCR4 have been reported to form a heteromeric receptor complex involved in MIF endocytosis, and colocalization of CD74 and CXCR4 has been observed [26]. Based on our results described above, we were interested in the possibility of the involvement of CD74 and CXCR4 colocalization in MIF regulation of monocyte adhesion to endothelial cells. Here, we observed both CD74 and CXCR4 by fluorescence microscopy. The results of immunofluorescence staining indicated that there was no significant enhancement in the surface expression of CD74. In contrast to CD74, the expression of CXCR4 was increased significantly by*P. gingivalis*. The results of IF staining showed the colocalization of CD74 and CXCR4 (Fig. 4).

### Receptor Complex Formation Between CD74 and CXCR4 for MIF in EA.hy926 Cells Infected with *P. gingivalis*

**Fig. 5:**
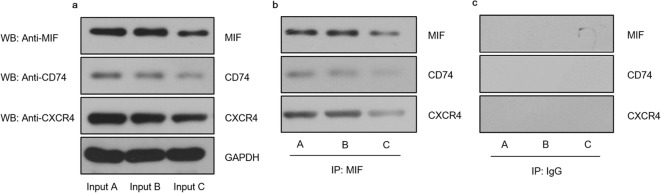
Receptor complex formation between CD74 and CXCR4 for MIF in EA.hy926 cells infected with *P. gingivalis*. Input controls (lysates without immunoprecipitation) are shown (**a**). Coimmunoprecipitation and pull-down assays were performed by anti-MIF antibody (**b**) or anti-IgG antibody (**c**) for immunoprecipitation (IP), and then anti-MIF antibody, anti-CXCR4 antibody, and anti-CD74 antibody were used for Western blot (WB). A: EA.hy926 cells infected with*P. gingivalis* for 24 h (MOI = 100). B: EA.hy926 cells stably transfected with empty vectors and infected with *P. gingivalis*. C: EA.hy926 cells stably transfected with *CXCR4-*silencing plasmid and infected with *P. gingivalis*. Data were representative of three independent experiments.

To verify whether CD74 and CXCR4 bind together to form a receptor complex in endothelial cells infected with *P. gingivalis*, coimmunoprecipitation and pull-down assays were performed. When lysates of EA.hy926 cells infected with *P. gingivalis* were immunoprecipitated with an anti-MIF antibody, a subsequent Western blot assay showed that CD74 and CXCR4 coprecipitated. In contrast, when lysates of endothelial cells stably transfected with the*CXCR4*-silencing plasmid were immunoprecipitated with the anti-MIF antibody, the levels of CD74, CXCR4, and MIF proteins were markedly decreased compared to the corresponding control (Fig. 5). Thus, in line with the suggestion that the level of ICAM-1 in endothelial cells and monocyte-endothelial cell adhesion induced by *P. gingivalis* infection is regulated by MIF, we present the hypothesis that portions of CD74 and CXCR4 may bind to form a receptor complex for MIF in endothelial cells infected with *P. gingivalis*. This interaction was further confirmed by the results of the fluorescence colocalization analysis and coimmunoprecipitations/pull-down experiments.

## DISCUSSION

MIF has been demonstrated to be closely associated with the progression and severity of atherosclerosis [17]. Animal experiments have shown that MIF is correlated with the thickening of the aortic intima and lipid deposition in mice and in rabbits fed an atherogenic diet [27, 28]. In contrast, *Mif* blockade in mice results in a regression of plaque areas [29]. Furthermore, antibody inhibition and genetic deletion studies have revealed that MIF influences the promotion of atherosclerosis by enhancing macrophage and T cell recruitment [21, 30].

*P. gingivalis* is considered to be a significant pathogen of periodontal disease and has been demonstrated to participate in the development of atherosclerosis. Interestingly, *P. gingivalis* DNA has been detected in atheromatous plaques [6, 31, 32]. Experiments in low-density lipoprotein (*Ldlp*)- and apolipoprotein E (*Apoe*)-deficient mice showed that *P. gingivalis* infection promotes atherosclerosis by markedly increasing lesion size and disease progression, which is followed by endothelial function impairment and systemic inflammation [3, 33]. Our early *in vitro* studies ascertained that *P. gingivalis* infection enhances the level of ICAM-1 in EA.hy926 cells and THP-1-EA.hy926 cell adhesion, demonstrating that *P. gingivalis* contributes to pro-atherosclerotic changes in endothelial cells [20]. Furthermore, we observed that *P. gingivalis* infection promoted MIF secretion in endothelial cells and that MIF was involved in the atherosclerotic plaque formation induced by *P. gingivalis* [21]. Currently, our understanding of the mechanisms by which*P. gingivalis* facilitates endothelial adhesion molecule expression and monocyte-endothelial cell adhesion is limited.

The functional receptor(s) of MIF and the molecular modes underlying its role in inflammatory diseases have remained elusive for decades. Recently, as a result of identifying CD74, CXCR4, and CXCR2 as receptors for MIF, we have gained a better understanding of the molecular mechanisms involved in MIF-mediated signaling pathways [29]. Depending on the cell type and its associated receptor expression profile, the activation of MIF is mediated by the different receptors [22, 25]. CD74 is a protein that participates in the formation and transport of MHC class II proteins and lacks signal-transducing intracellular domain [29, 34–36]. MIF binds to the extracellular domain of CD74 to form a ligand-receptor complex that activates multiple signaling pathways to participate in inflammatory responses [34, 35]. CXCR2 and CXCR4 may serve as additional signal transduction receptors of CD74 in MIF-stimulated inflammatory response [22, 29]. CXCR2 and CXCR4 are CXC chemokine receptors, and both of them belong to G protein-coupled receptor family [37, 38]. The colocalization of CXCR2 and CD74 in the cell membrane has been observed [23, 29]. The interaction between CXCR2 and CD74 suggests that MIF may affect downstream signal transduction through functional CXCR/CD74 complexes. Simons et al. found that MIF-mediated chemotaxis of endothelial progenitor cells through CXCR4 [39]. It was observed that MIF activates JNK signaling and upstream kinases PI3K and Src by binding to CXCR4 and CD74, thereby regulating inflammation, cell differentiation, and apoptosis [22]. Schwartz et al. confirmed that CXCR4 and CD74 colocalize in the cell membrane, forming a functional receptor complex to mediate MIF-specific signal transduction [26, 40]. Thus, this evidence indicates that functional CD74/CXCR receptor complexes may occur. However, in the presence of *P. gingivalis*, the specific receptor(s) or receptor complex of MIF have not yet been defined.

In the current study, we focused on the combination of CD74, CXCR4, and MIF. By analyzing the receptor protein expression, we showed that the expression of CXCR4 was significantly enhanced in endothelial cells infected with *P. gingivalis*, independent on CD74 co-expression. In contrast, both CD74 and CXCR4 were shown to be involved in the increased ICAM-1 levels in EAhy.926 cells and in the observed enhanced THP-1-EAhy.926 cell adhesion. Thus, we present the hypothesis that CXCR4 and CD74 form a receptor complex that is responsive to MIF. First, through fluorescence microscopy observations, we demonstrated that CD74 and CXCR4 colocalized at the surfaces of infected endothelial cells. Subsequently, we investigated whether CD74 interacts with CXCR4 in endothelial cells infected with *P. gingivalis*. The co-immunoprecipitation and pull-down analyses were performed using stably transfected EA.hy926 cells with the *CXCR4*-silencing plasmid and an MIF antibody. Our results provided a hint to the functional interplay between CD74 and CXCR4, revealing that CD74 and CXCR4 form a receptor complex that bind to MIF in infected EA.hy926 cells. We speculate that MIF activates monocyte adhesion to the vascular endothelial cell surface, promoting plaque formation associated with atherosclerotic disease by interacting with CD74 and CXCR4. Furthermore, it is conceivable that CD74/CXCR4 complexes play a crucial part in *P. gingivalis*-induced atherogenic lesion formation. Interestingly, we also found that when the CXCR4 gene was knocked down, it seemed that the expression of MIF decreased to some extent. We hypothesized that due to the redundant unbound MIF by CXCR4 gene silence, some uncertain negative feedback might be activated, resulting in the inhibition of MIF synthesis. This potential association between MIF and CXCR4 requires our further experiments to confirm.

Overall, the results of our study indicated that the CD74/CXCR4 complex was responsive to MIF target cell activation upon *P. gingivalis* infection. However, it has been reported that CD74 can also be combined with other proteins, such as CXCR2 in signal transduction of MIF. In addition, it is suggested that blocking the signal transduction of MIF can be used as a therapeutic target in inflammatory diseases, such as atherosclerosis. Considering the multiple receptors of MIF, we believe that a combination of blocking each receptor may achieve better therapeutic effects. Therefore, additional experiments are needed to identify other proteins bound to CD74 or CXCR4 during the*P. gingivalis* infection process.

## CONCLUSION

Our experiments revealed that CD74 and CXCR4 are involved in the pathological processes by which *P. gingivalis* promotes ICAM-1 expression and monocyte-endothelial cell adhesion. MIF-CD74-CXCR4 ligand-receptor complexes were present in endothelial cells infected with *P. gingivalis*, which may play a significant role in*P. gingivalis*-induced atherosclerosis.

## Abbreviations

*P. gingivalis*: *Porpyromonas gingivalis*
MIF: macrophage migration inhibitory factor
ICAM-1: intercellular adhesion molecule-1
DMEM: Dulbecco’s modified Eagle medium
FBS: fetal bovine serum
MOI: multiplicity of infection
qRT-PCR: quantitative real-time polymerase chain reaction
